# tDCS randomized controlled trials in no-structural diseases: a quantitative review

**DOI:** 10.1038/s41598-021-95084-6

**Published:** 2021-08-11

**Authors:** Eugenia Gianni, Massimo Bertoli, Ilaria Simonelli, Luca Paulon, Franca Tecchio, Patrizio Pasqualetti

**Affiliations:** 1 LET’S – ISTC – CNR, via Palestro 32, 00185 Rome, Italy; 2 Unit of Neurology, Neurophysiology, Neurobiology, Department of Medicine, University Campus Bio-Medico of Rome, Rome, Italy; 3 Department of Imaging and Neuroscience and Clinical Sciences, University ‘G. D’Annunzio’ of Chieti-Pescara, Chieti, Italy; 4 Service of Medical Statistics and Information Technology, Fatebenefratelli Foundation for Health Research and Education, Rome, Italy; 5 Department of Public Health and Infectious Diseases, Section of Medical Statistics, University of Rome “Sapienza”, Rome, Italy

**Keywords:** Neuroscience, Diseases of the nervous system, Therapeutics

## Abstract

The increasing number and quality of randomized controlled trials (RCTs) employing transcranial direct current stimulation (tDCS) denote the rising awareness of neuroscientific community about its electroceutical potential and opening to include these treatments in the framework of medical therapies under the indications of the international authorities. The purpose of this quantitative review is to estimate the recommendation strength applying the Grading of Recommendations, Assessment, Development and Evaluations (GRADE) criteria and PICO (population, intervention, comparison, outcome) model values for effective tDCS treatments on no-structural diseases, and to provide an estimate of Sham effect for future RCTs. Applying GRADE evaluation pathway, we searched in literature the tDCS-based RCTs in psychophysical diseases displaying a major involvement of brain electrical activity imbalances. Three independent authors agreed on Class 1 RCTs (18 studies) and meta-analyses were carried out using a random-effects model for pathologies sub-selected based on PICO and systemic involvement criteria. The meta-analysis integrated with extensive evidence of negligible side effects and low-cost, easy-to-use procedures, indicated that tDCS treatments for depression and fatigue in Multiple Sclerosis ranked between moderately and highly recommendable. For these interventions we reported the PICO variables, with left vs. right dorsolateral prefrontal target for 30 min/10 days against depression and bilateral somatosensory vs occipital target for 15 min/5 days against MS fatigue. An across-diseases meta-analysis devoted to the Sham effect provided references for power analysis in future tDCS RCTs on these clinical conditions. High-quality indications support tDCS as a promising tool to build electroceutical treatments against diseases involving neurodynamics alterations.

## Introduction

The recently coined term ‘*electroceuticals*’^1,2^, the cure of pathologies by electrical signals, has opened up to new therapeutic strategies when the body-brain system suffers secondary to neuronal activities unbalances^3^ by externally inducing electrical inputs to restore them. *Electroceuticals* refers to a sector full of great expectations regarding its therapeutic potential^4^, exploiting the great advancements within technological, conceptual and computing fields. On one side, there is an increasingly clear understanding of the body-brain system as a multidimensional network^5^ where the neuronal electrical transmission sustains the communication among different nodes, in turn interacting with the functionality of the system at multiple levels (immune, behavioural and hormonal)^6,7^. On the other side, there is an acknowledgement that it is possible to communicate more effectively and by means of increasingly miniaturized and skilled devices with the body-brain network also by sending appropriate signals directly to some specific nodes of the control network, strengthening bridges where communication has gone depleted due to a pathological condition^8,9^. A relevant branch of electroceuticals is the non-invasive brain stimulation (NIBS), where the two main techniques are repetitive transcranial magnetic stimulation (rTMS)^10^ and transcranial direct current stimulation (tDCS)^11^. Even though there is extensive and robust evidence of the clinical effectiveness of the use of rTMS in multiple pathologies^10^ our purpose is to focus here on the use of tDCS since we think that, while rTMS is more suited for high intensity and focused on small cortical areas stimulations, tDCS displays its benefits when focused on wider cerebral areas and usually delivers small currents. We believe that no-structural diseases, i.e. the focus the present review, may mostly benefit from a tDCS treatments as long as they involve electrical activity unbalances that can spread on wider cortical areas and be treated superficially by small currents.

tDCS consists in the extracranial delivery of a weak direct electric current that flows from the anode to the cathode, with an overall effect of increased excitability in areas below the anode (depolarization) and decreased excitability in correspondence of the cathode (hyperpolarization)^12^.

There is a large bulk of evidence that single tDCS stimulation of 10–20 min in healthy subjects induces long-lasting effects on multiple behavioural and cognitive functions, such as memory^13^, motor control^14^, attention^15^, perception^16^. Moreover, experience indicates multiple session treatments as safe^17^ and producing effects lasting from weeks to months^18^. In the very last years, motivated by a large bulk of descriptive investigations with promising results, the international community started tDCS randomized controlled trials (RCTs)^19,20^.

Here, focusing on tDCS, we questioned the literature on these RCTs proceeding in agreement with the PICO framework -Population, Intervention, Comparison, Outcome- of the Grading of Recommendations, Assessment, Development and Evaluations (GRADE) of clinical practices^21^ (Table 1) which presents a well-accepted methodology for framing health care questions endorsed by the Cochrane collaboration for EBM (evidence-based medicine) assessments. We selected populations affected by symptoms secondary to alterations of neuronal electrical activity, expected as the population benefitting the most from the extracted recommendations.

**Table 1:** Quality of recommendation of nonpharmacologic treatments.

Rating criteria of Treatments
Balance between desirable and undesirable outcomes (trade-offs) taking into account: - best estimates of the magnitude of effects on desirable and undesirable outcomes - importance of outcomes (estimated typical values and preferences)
Confidence in the magnitude of estimates of effect of the interventions on important outcomes (overall quality of evidence for outcomes)
Confidence in values and preferences and their variability
Resource use

The Treatment Rating criteria from GRADE.

We pursued a triple aim: 1. to estimate the GRADE recommendation strength of tDCS against symptoms sustained by neuronal electrical activity unbalances. To be inclusive at most, i.e. to identify all domains where tDCS has been used for clinical purposes, we indicated, instead of the list of pathologies to include, the structural pathologies to be excluded. In assessing the clinical efficacy of the interventions, we applied a more conservative selection, considering only GRADE Class 1 RCTs, and submitted multiple studies on the same pathology to a meta-analysis quantitative assessment. We reported all those RCTs independently of the level of efficacy: this choice was made in order to give a quantification of the expected impact of the treatment and to evaluate it in relationship with the stimulation parameters or population features; 2. to indicate the PICO variables values for effective tDCS treatments, i.e. the clinical conditions most likely to benefit, with which stimulation parameters and with which outcome measures; 3. a further contribute of this quantitative review is the estimation of Sham effect in trials planned to assess the efficacy of tDCS. This issue is relevant for the following reasons: even if there are several papers dealing with estimation of placebo effect and a Cochrane publication in 2010^22^ tempered down its clinical relevance, we considered that the peculiar experimental setup of non-invasive brain stimulation could exert “per se” a valuable effect on subjective assessments, such as mood status and perceived fatigue. Furthermore, the quantitative estimation of Sham effect could be useful to plan future clinical trials both when the arm with Sham stimulation would be considered and when, for feasibility and/or ethical reasons, only the arms with Real stimulations would be designed. We attempted to provide a support to guide power analysis and computation of the appropriate sample size of incoming RCTs.

## Methods

### Eligibility criteria for studies’ search and selection

#### Selection of PICO variables

Participants were adults with no-structural diseases. We excluded patients with stroke, dementia, Alzheimer or Parkinson disease, palsy. The intervention was limited to tDCS with clearly stated montage, delivered current and session durations. The comparison condition was always the Sham treatment. The outcome measure was a selection criterion for studies submitted to the meta-analyses.

#### Quality of treatments’ recommendation

**Figure 1:**
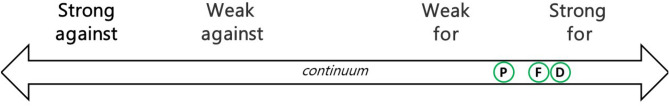
Treatment recommendation strength. The strength of a recommendation of a clinical procedure ranges in a continuum divided into categories and reflects the extent to which a guideline panel is confident that desirable effects outweigh undesirable effects. The results of the present meta-analysis indicate depression (D), fatigue in multiple sclerosis (F), and pain (P) as conditions in which the GRADE recommendation of tDCS treatments ranges between moderate and strong.

Since interested in a review of the tDCS applications with clinical relevance, we relied on the competent regulatory authorities who published the GRADE of medical treatments^21^ to outline the Treatment Rating Criteria (Table 1) for assigning the position of a procedure within a continuum of recommendation strength ranging from ‘strong against’ to ‘strong for’ (GRADE Chapter 6, Going from evidence to recommendations, Fig. 1).

Aware that GRADE recommendations should never be viewed as dictates, we followed a key criterion that is that the evidence in support of the treatment use is of high quality. In this respect, we selected solely the RCTs in Class 1, defined as satisfying all the criteria listed in Table 2 RCT classification, according to the methods’ section of the CONSORT (Consolidated Standards of Reporting Trials) checklist for RCTs of non-pharmacologic treatments^23^.

**Table 2:** Classification criteria of RCTs.

Sample size estimate to enrol adequately powered groups
Control condition
Randomization assignment
Masking
Randomization concealment
Primary outcome clearly defined
Exclusion/inclusion criteria clearly defined
Adequate accounting for dropouts and crossovers with numbers sufficiently low to have minimal potential for bias
Relevant baseline characteristics are presented and substantially equivalent among treatment groups or there is appropriate statistical adjustment for differences

The RCT classification criteria from CONSORT.

#### Electronic repository search strategy

We entered in *PubMed* the following search query and keywords: Filters: Randomized Controlled Trial, Search: ((((((tDCS transcranial direct current stimulation) NOT stroke) NOT dementia) NOT Alzheimer) NOT Parkinson) NOT palsy) NOT “brain injury” AND later than Sept 2016. By applying these criteria, we got a list of 266 articles. Among these articles, based on the title we excluded articles concerning non-RCT and articles concerning structural pathologies not excluded by our first search. Studies on animals were excluded. The articles published before 2016 were taken from Leufaucher et al.^24^ who reviewed tDCS RCTs published up to September 2016, applying our selection criteria. Reading the papers, we excluded those non satisfying the RCT Class1 criteria. To apply the criterion of ‘adequately powered’ we applied the search (size OR sample OR power).

#### Study selection

Three review authors (EG, MB and IS) independently obtained and assessed potentially eligible articles for inclusion in the review. Any disagreements were resolved through discussion with the author FT.

For multiple publications from the same trial, we considered only one data set, the most recent or the bigger one.

### Meta-analysis design

#### Data collection

When the inclusion criteria led to multiple studies for a pathology, we executed a meta-analysis to assess the dimension of the effect.

From each study included in the meta-analysis, we extracted the number of participants randomized and analysed in each treatment group (Real, Sham). Since the outcomes were continuous, we collected the scale used to measure intervention effect, mean and standard deviation (SD) or standard error pre- and post-treatments when reported. If baseline and post intervention data were not reported, it was extracted from graph by *graphreader.com*. The percentage mean variation and standard deviation were extracted.

For one study^25^ data about responders and non-responders were combined.

If more longitudinal measurements of the outcome were reported, we considered the first.

#### Quantitative treatment description—Summary measures

In agreement with PRISMA procedures^26^, we quantified the effect of each treatment, Sham and Real tDCS, calculating the effect size and the relative Standard Error (SE), applying the formulas reported in Borenstein, Hedges, Higgins, Rothstein (2009), Chapter 4^27^.

Difference between mean change scores observed in Sham and in Real tDCS groups was analyzed, the summary statistics used was the standardized mean difference (SMD) because of some studies using different scales of measurement. The SMD was calculated following the indication in Chapter 6 of Cochrane handbook^28^. To apply the formulas, we needed the correlation between pre-intervention and post-intervention data, when none of the studies reported it, we calculated from data available. Subsequently a sensitivity analysis was performed considering a correlation of 0.5.

We applied a random-effect meta-analysis. We presented all effects with 95% Confidence Interval (CI). ES (SMD) values were interpreted as: 0.2 represents a small effect, 0.5 a moderate effect and 0.8 a large effect^29^.

#### Assessment of heterogeneity

The heterogeneity was evaluated via the Cochran’s Q test and quantified through the I^2^. The I^2^ describes the rate of variation across studies due to heterogeneity rather than chance, ranging from 0 (no heterogeneity) to 100 (maximal heterogeneity).

#### Risk of bias across studies

Funnel plot was used to investigate the presence of publication bias.

### PICO variables’ values of efficacious tDCS treatments

Based on indications of meta-analyses about the treatment efficacy in the diverse clinical conditions, we provided a qualitative review of the specific protocols applied. For each of the pathologies included in the quantitative analysis, we introduced a schematic representation of the PICO variables, with the inclusion criteria defining the population of interest, the outcome measure, the tDCS montage, delivered current amplitude and stimulation duration. In particular, we underlined the superficial current density as the key physical parameter to be modulated, together with the total amount of delivered current.

### Ethics and patient consent

Our study did not require an ethical board approval because this study retrieved and synthesized data from already published studies.

## Results

### Methods and selection flowchart

**Figure 2:**
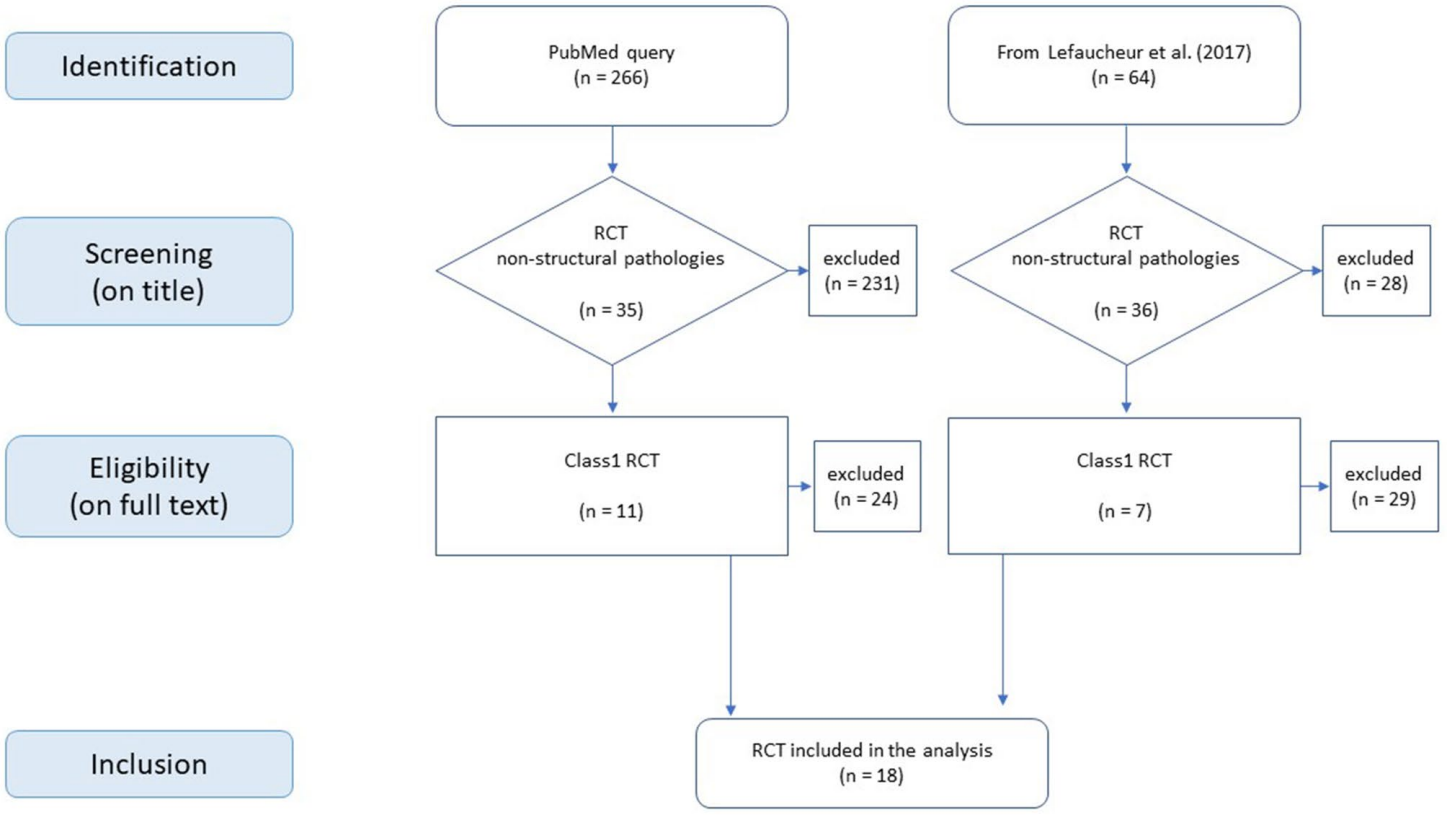
Flow chart. PRISMA flow diagram illustrating search strategy, inclusion and exclusion process.

By the search and selection process depicted in the flowchart (Fig. 2), we started from 330 arriving to 18 analysed RCT papers (4 depression, 4 fatigue in MS, 1 pain, 5 addictions, 4 fibromyalgia).

#### Overview of the Class 1 tDCS RCT in no-structural diseases

We reported a description of all the non-structural studies organized by pathology. We started with those for which the meta-analysis was executed and we reported a qualitative description for those excluded for the meta-analysis, clarifying the reason for this choice.

For each pathology, we quantified the efficacy of the treatment for Sham, Real their relationship, and eventually specified the population enrolment criteria, the outcome measure and stimulation parameters.

### Depression

#### Meta-analysis

**Figure 3:**
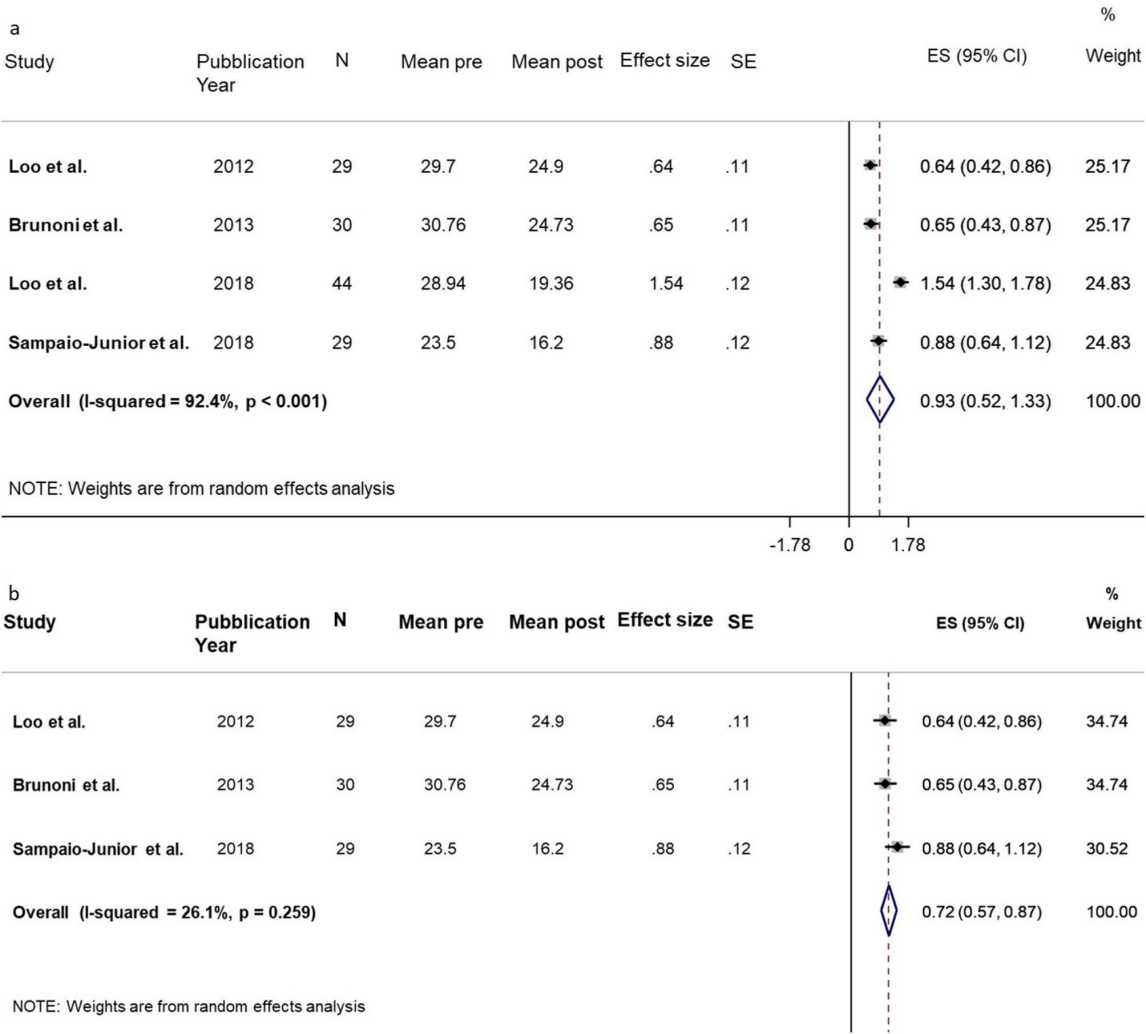
Depression. Sham. (**a**) Forest plot of meta-analysis results considering all studies. The diamond represents the pooled standardized mean difference (SMD, dashed red vertical line) and its 95% confidence interval; vertical solid dark line is the line of equivalence. The estimates for each study and their 95% confidence intervals are represented by a box with whiskers, the dimension of grey box is proportional to the precision of the study. (**b**) Forest plot of sensitivity meta-analysis. Note: SE = Standard Error; ES = Effect Size. CI = Confidence Interval.

**Figure 4:**
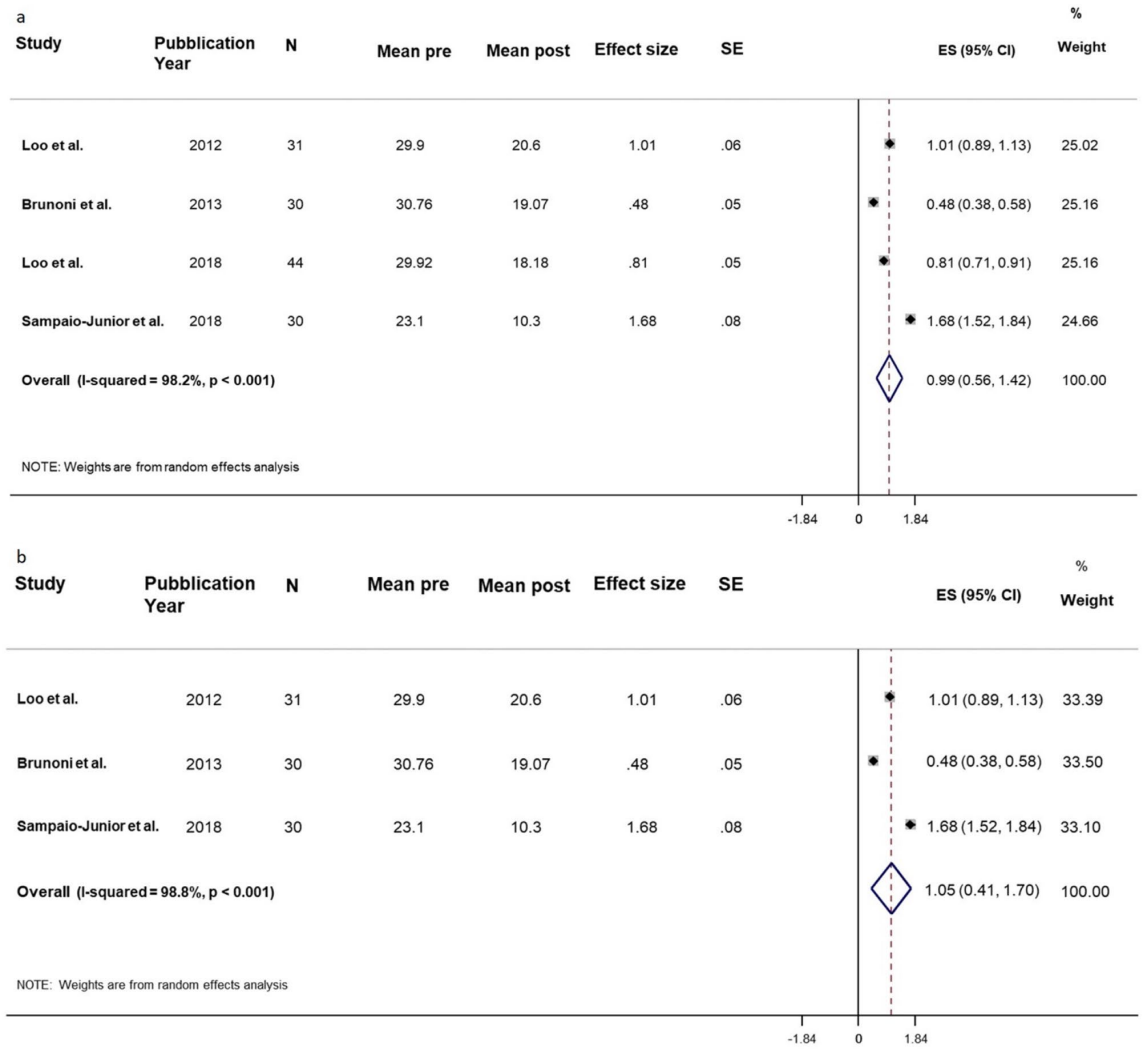
Depression. Real. (**a**) Forest plot of meta-analysis results considering all studies. The diamond represents the pooled standardized mean difference (SMD, dashed red vertical line) and its 95% confidence interval; vertical solid dark line is the line of equivalence. The estimates for each study and their 95% confidence intervals are represented by a box with whiskers, the dimension of grey box is proportional to the precision of the study. (**b**) Forest plot of sensitivity meta-analysis. Note: SE = Standard Error; ES = Effect Size. CI = Confidence Interval.

One study^30^ reported the percentage mean and standard deviation (SD) variation but none of the studies reported the mean change score and the SD, data were calculated as described in statistical method section. The correlation (Pearson’s r) calculated from data was equal to 0.86 in Sham group (Table 3, Fig. 3) and to 0.96 in Real group (Table 4, Fig. 4).

**Table 3:** Depression. Sham.

Study	PY	Study designed	Scale	n	Mean pre	SD pre	Mean post	SD post	Mean Diff	SD Diff^a^	SD within^a^	Effect Size^a^ (SMD)	SE of effect size^a^	SD Diff^b^	SD within^b^	Effect Size^b^ (SMD)	SE of effect size^b^
Loo et al	2012	Parallel	MADRS	29	29.7	5.7	24.9	7.6	4.8	3.96	7.48	0.64	0.11	6.86	6.86	0.70	0.21
Brunoni et al	2013	Factorial Randomized Controlled	MADRS	30	30.8	5.3	24.7	8.7	6.03	4.90	9.26	0.65	0.11	7.56	7.56	0.80	0.21
Loo et al	2018	Parallel	MADRS	44	28.9	2.6	19.4	5.28	9.58	3.29	6.22	1.54	0.12	4.57	4.57	2.10	0.27
Sampaio-Junior et al	2018	Parallel	HDRS-17	29	23.5	4.7	16.2	7.7	7.3	4.37	8.27	0.88	0.12	6.72	6.72	1.09	0.23
**Pooled analysis**				**132**								**0.93**	**0.21**			**1.15**	**0.29**
**Pooled analysis without Loo et al. 2018**				**88**								**0.72**	**0.08**			**0.85**	**0.13**

Data about pre and post data in Sham group. PY: Pubblication year. SD: Standard Deviation. SMD = Standardized Mean Difference. SE = Standard Error.

Note: ^a^ Correlation pre-post r = 0.86. ^b^ Correlation pre-post r = 0.50.

**Table 4:** Depression. Real.

Study	PY	Study designed	Scale	n	Mean pre	SD pre	Mean post	SD post	Mean Diff	SD Diff^a^	SD within^a^	Effect Size^a^ (SMD)	SE of effect size^a^	SD Diff^b^	SD within^b^	Effect Size^b^ (SMD)	SE of effect size^b^
Loo et al	2012	Parallel	MADRS	31	29.7	5.7	20.6	7.6	9.3	2.6	9.24	1.01	0.06	6.84	6.84	1.36	0.25
Brunoni et al	2013	Factorial Randomized Controlled	MADRS	30	30.8	5.78	19.07	12.2	11.7	6.9	24.24	0.48	0.05	10.58	10.58	1.11	0.23
Loo et al	2018	Parallel	MADRS	44	29.9	1.96	18.18	5.9	11.7	4.1	14.47	0.81	0.05	5.24	5.24	2.24	0.28
Sampaio-Junior et al	2018	Parallel	HDRS-17	30	23.1	3.9	10.3	5.6	12.8	2.2	7.61	1.68	0.08	4.97	4.97	2.57	0.38
**Pooled analysis**				**135**								**0.99**	**0.22**			**1.78**	**0.33**
**Pooled analysis without Loo et al. 2018**				**88**								**1.05**	**0.33**			**1.62**	**0.38**

SD = Standard Deviation. SMD = Standardized Mean Difference. SE = Standard Error.

Note: ^a^ Correlation pre-post r = 0.96. ^b^ Correlation pre-post r = 0.5.

The Sampaio-Junior et al.’s study^31^, reported data about depression measured by Hamilton depression rating scale (HDRS-17) scores while the other three studies^30,32,33^ reported data about Montgomery-Asberg depression rating scale (MADRS). For one study^33^ data were extracted from graph.

Pooling the studies, the total number of patients in was 132 the Sham group and 135 in the Real group.

##### Depression—Sham effect

The pooling effect size was equal to 0.93 (95% CI 0.52–1.33; *p* < 0.001), indicating a large Sham effect. The heterogeneity was considerable (I^2^ = 92.4%, *p* < 0.001).

We performed a sensitivity analysis excluding the Loo et al. study (2018), because the data were extracted from the graph. The pooled effect size was equal to 0.72 SDs (95% CI 0.57–0.87; *p* < 0.001), indicating a moderate Sham effect. The heterogeneity was not significant (I2 = 26.1%, *p* = 0.259).

Another sensitivity analysis, performed decreasing the correlation to 0.5, showed consistent results about the reduction after sham (ES = 1.15, 95% CI 0.59–1.71; *p* < 0.001). The heterogeneity was considerable (I2 = 84.5%, *p* < 0.001). Reperformed the analysis excluding Loo et al. 2018, the pooled effect was reduced to 0.85 (95% CI 0.61 to 1.095; *p* < 0.001) and the heterogeneity inter trials was not significant (I2 = 0%, *p* = 0.437) (Table 3, Fig. 3).

##### Depression—real effect

The pooling effect size was equal to 0.99 (95% CI 0.56–1.42; *p* < 0.001), indicating a large effect after tDCS. The heterogeneity was considerable (I^2^ = 98.2%, *p* < 0.001).

We performed a sensitivity analysis excluding the Loo et al.’s study 2018, because the data were extracted from the graph.

The pooled effect size was equal to 1.05 (95% CI 0.41–1.70; *p* = 0.001), indicating a large effect after tDCS. The heterogeneity was considerable (I^2^ = 98.8%, *p* < 0.001).

The elevate heterogeneity was maybe due to different administration condition of tDCS.

Another sensitivity analysis, performed decreasing the correlation to 0.5, showed consistent results about the reduction after tDCS (ES = 1.78, 95% CI 1.13–2.43; *p* < 0.001). The heterogeneity was considerable (I^2^ = 82.5%, *p* = 0.001) (Table 4, Fig. 4).

##### Depression—Real vs Sham effect

**Figure 5:**
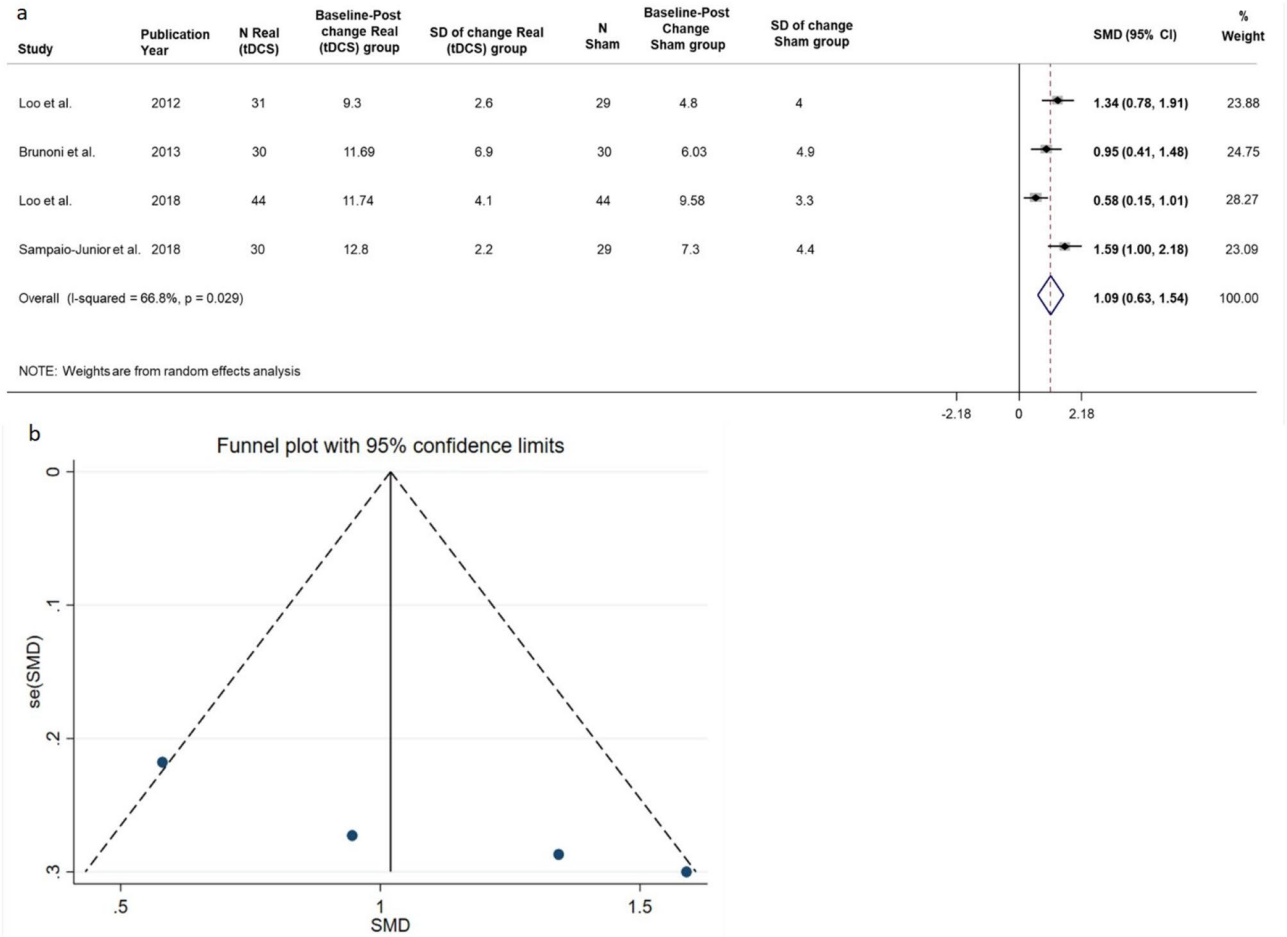
Depression. Real Vs Sham. (**a**) Forest plot of meta-analysis results considering all studies. The diamond represents the pooled standardized mean difference (SMD, dashed red vertical line) and its 95% confidence interval; vertical solid dark line is the line of equivalence. The estimates for each study and their 95% confidence intervals are represented by a box with whiskers, the dimension of grey box is proportional to the precision of the study. (**b**) On y-axis is represented the standard errors of the studies from lowest to higher, on x-axis the estimated SMDs; the solid vertical line represents the pooled SMD and diagonal dashed line represents its 95% CI.

The results showed that the tDCS had a large effect, the reduction in depression observed after tDCS was on average 1.09 SDs higher than that observed after sham (95% CI 0.63–1.54; *p* < 0.001) (Table 5). Heterogeneity was moderate and significant (I2 = 66.8%, *p* = 0.029) (Fig. 5).

**Table 5:** Depression. Real Vs Sham.

				Baseline-post mean change						95% CI		
Study	PY	Study designed	Scale	N Sham	N Real	Sham group	SD Sham group	Real group	SD Real group	SMD	LL	UL
Loo et al	2012	Parallel	MADRS	29	31	4.8	4	9.3	2.6	1.34	0.78	1.91
Brunoni et al	2013	Factorial, Randomized, Controlled	MADRS	30	30	6.03	4.9	11.69	6.9	0.95	0.41	1.48
Loo et al	2018	Parallel	MADRS	44	44	9.58	3.3	11.74	4.1	0.58	0.15	1.01
Sampaio-Junior et al	2018	Parallel	HDRS-17	29	30	7.3	4.4	12.8	2.2	1.59	1.00	2.18
**Pooled analysis**										**1.09**	**0.63**	**1.54**

SD = Standard Deviation.SMD = Standardized Mean Difference. CI = Confidence Interval. LL = Lower Limit. UL = Upper Limit.

By the funnel plot no evidence of publication bias emerged. We performed a sensitivity analysis excluding the Loo et al.’s study 2018, for the reason previously explained. The pooled effect size was equal to 1.28 SDs (95% CI 0.91–1.65; *p* < 0.001), indicating a large effect after tDCS. The heterogeneity was not significant (I^2^ = 23.5%, *p* = 0.271).

The sensitivity analysis, considering a correlation of 0.5, showed that the results were consistent. The reduction in depression observed after tDCS was significantly higher than that observed after Sham (SMD = 0.63, 95% CI 0.39–0.88; *p* < 0.001). The heterogeneity was not significant (I^2^ = 0%, *p* = 0.570).

### PICO variables’ values for tDCS against depression

**Figure 6:**
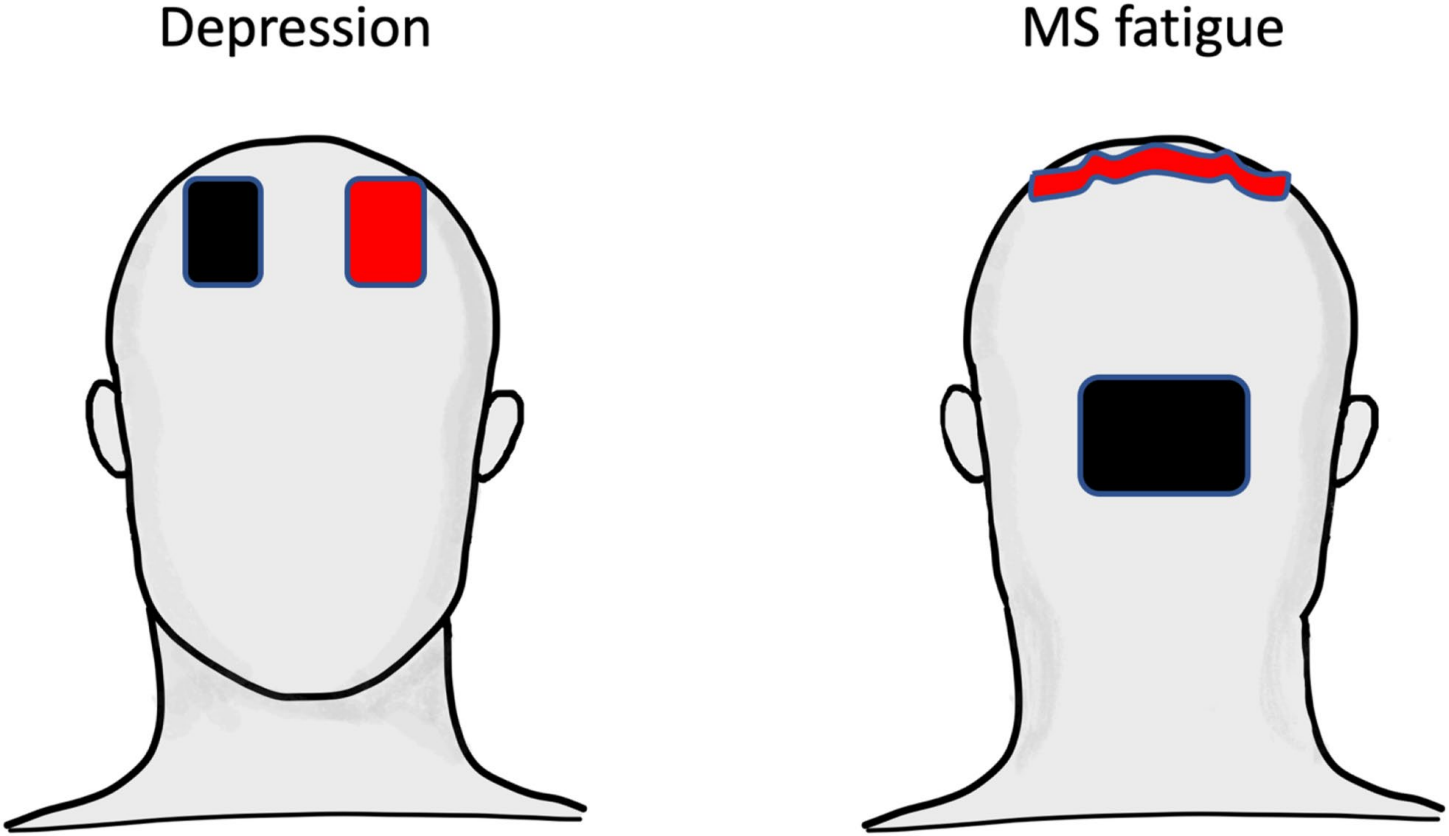
tDCS montage for depression and MS fatigue. Graphical representation of the tDCS electrodes position and shape. In Depression the rectangular (7 × 5 cm^2^) anode (red) is centred on F3 and the cathode (black) on F4. In MS fatigue, the anode is an electrode with 35 cm^2^ area shaped as the individual central sulcus cortical folding and the occipital cathode a double area rectangle (7 × 10 cm^2^).

The depressed patients populations who benefit by tDCS suffer by moderate to severe symptoms (Table 6). Data suggest the use of MADRS as outcome measure, since it was used by all but one study, which employed HDRS as primary outcome and observed similar results when assessed by MADRS. When defining the most efficacious tDCS intervention parameters, we observed that all studies position the anode on left dorsolateral prefrontal cortex (dlPFC), targeted according to the 10–20 international system of EEG electrodes placement by centering the anode on F3. Concerning the cathode, F8 seems to drive the most stable result, since the two studies using F4 instead obtained the best and worst result. Nevertheless, the results from F8 cathode position are in-between, so that we suggest that there is no advantage in making the position of anode and cathode asymmetrical in the two hemispheres. We therefore suggest the montage of anode on F3, cathode ﻿on F4 (Fig. 6).

**Table 6:** Parameters. Depression.

Study	Outcome	Population	Intervention						
			Electrode position		Stimulation intensity			Duration	
			Anode	Cathode	Electrode size	Ci	Csd	Daily	Days
Loo 2012	MADRS	MADRS > 20	F3	F8	35/35	2	0.057	20	15
Brunoni 2013	HDRS	HDRS > 17	F3	F4	25/25	2	0.080	30	10
Loo 2018	MADRS	MADRS > 20	F3	F8	35/35	2.5	0.071	30	20
Sampaio 2018	MADRS	MADRS > 20	F3	F4	25/25	2	0.080	30	10

PICO variables for tDCS against depression. The parameters of tDCS intervention include the electrodes’ position (El position) expressed by the site of the 10–20 EEG International System where the electrode was centered; the stimulation intensity considering the area of the electrodes (El size, cm^2^) for anode/cathode, the current intensity (Ci, mA), and the current superficial density (Csd, mA/ cm^2^); the stimulation duration with the daily session duration (min) and the number of days (Days).

In relation to the current delivered, it varies little between the studies and the higher current surface density (CSD) used in two studies, results in the best and the worst efficacy, so the suggested csd can be the average across the four studies, around to 0.072 mA/ cm^2^.

In relation to the duration, 10 days of treatment seem sufficient, in fact the best result is with 10 days (Sampaio et al.), and Loo and colleagues observed a lower result with 20 days treatment than the 15 days one. We suggest the most used daily session duration of 30 min, even though Loo et al. with 20 min duration observed a result comparable to 30 min one. Therefore, suggested duration is 30 min/day, 10 days.

In conclusion, given that rTMS has been accepted by FDA as a clinical treatment^34^ since displaying evident and robust benefits against this pathology, it would be interesting to evaluate whether tDCS may be applied in the immediately preceding phases of the manifestation of major depression.

### MS fatigue

#### Meta-analysis

Five studies^25,35–37^ fulfilled the criteria of class 1 studies. Tecchio et al.’s 2015 study considered the same sample of Tecchio et al. 2014 for the S1 target, so only data of the most recent study was considered in the meta-analysis. The group stimulated on hand SM1 in Tecchio et al. 2015 was not considered since it was included in Ferrucci et al.’s study, which involved a larger group (Table 4, 7, 10, Figs. 4, 6, 8).

**Table 7:** Fatigue. Sham.

Study	PY	Study designed	Scale	n	Mean pre	SD pre	Mean post	SD post	Baseline-post mean change	SD Diff	Effect size ^a^ (SMD)	SE of effect size ^a^
Tecchio et al	2015	Crossover	MFIS	13	37	7	35	10	– 3	4	0.17	0.08
Cancelli et al	2018	Crossover	MFIS	10	51	12	46	19	- 5	8	0.20	0.09
Ferrucci et al	2014	Crossover	FIS	23	111	42	96	42	- 15	12	0.36	0.06
**Pooled analysis**											**0.27**	**0.08**

PY = Publication Year. SMD = Standardized Mean Difference. SE = Standard Error.

Note: ^a^ Correlation pre-post r = 0.96 was assumed to calculate the effect size and the corresponding standard error.

Since all the trials were cross-over, methodological methods indicated by Elbourne et al.^38^ were followed. As measure of correlation between the two conditions (Sham and Real), the coefficient indicated by one of the individuated studies (Cancelli et al., r = 0.55) was assumed. The summary statistics used was the standardized mean difference (SMD). We calculate the effect size (ES) and the relative Standard error (SE) as describe in method-summary measures section.

Pooling the studies, the total number of patients was 46 subjects.

##### Ms Fatigue–Sham effect

**Figure 7:**
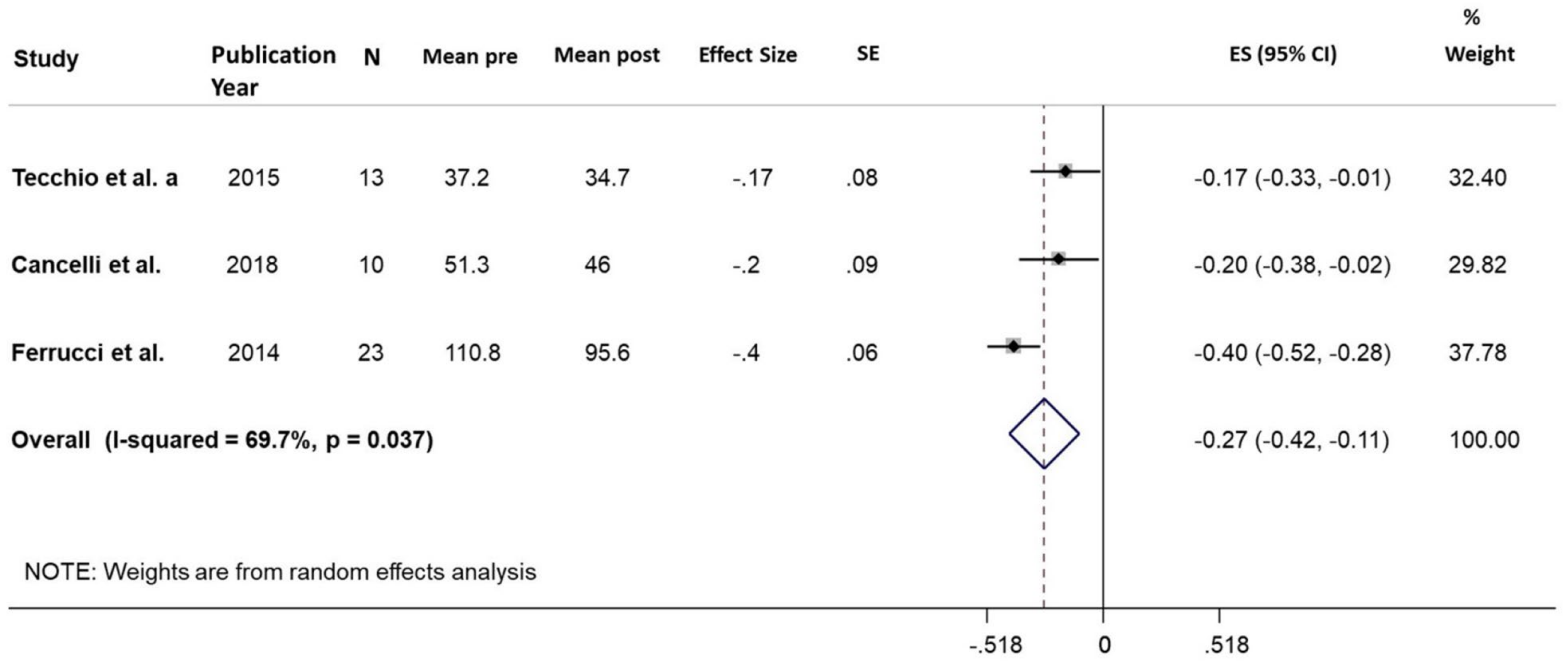
MS Fatigue. Sham. Forest plot of meta-analysis results considering all studies. The diamond represents the pooled standardized mean difference (SMD, dashed red vertical line) and its 95% confidence interval; vertical solid dark line is the line of equivalence. The estimates for each study and their 95% confidence intervals are represented by a box with whiskers, the dimension of grey box is proportional to the precision of the study. Note: SE = Standard Error; ES = Effect Size. CI = Confidence Interval.

The pooled ES indicated a significant small effect of the sham (ES = 0.27, 95% CI 0.11 to 0.42; *p* = 0.001). The Heterogeneity was moderate (I^2^ = 69.7%, *p* = 0.037). One possible source of Heterogeneity was the Electrode positions. We reperformed meta-analysis considering only two studies with the same Electrode positions (Tecchio et al.^36^ and Cancelli et al.^37^). The results confirmed what seen previously, a significant small effect of Sham (ES = 0.18, 95% CI 0.07 to 0.3; *p* = 0.002). The Heterogeneity was not significant (I^2^ = 0%, *p* = 0.803) (Table 7, Fig. 7).

##### Ms Fatigue–Real effect

**Figure 8:**
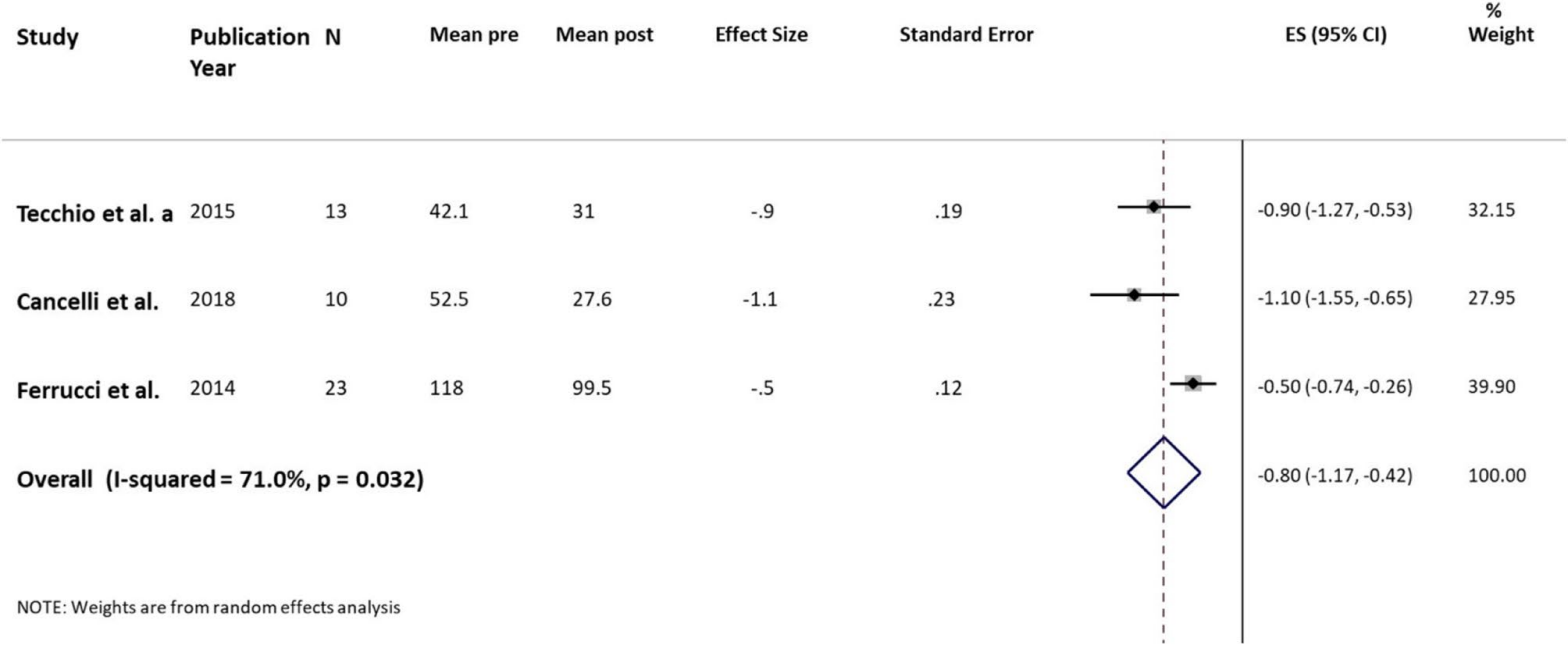
MS Fatigue. Real. Forest plot of meta-analysis results considering all studies. The diamond represents the pooled standardized mean difference (SMD, dashed red vertical line) and its 95% confidence interval; vertical solid dark line is the line of equivalence. The estimates for each study and their 95% confidence intervals are represented by a box with whiskers, the dimension of grey box is proportional to the precision of the study. Note: SE = Standard Error; ES = Effect Size. CI = Confidence Interval.

We calculate the Standardize Mean Difference as effect size (ES) and the relative Standard error (SE) as describe in method-summary measures section. The pooled ES indicated a significant large effect of Real (tDCS) (ES = 0.80, 95% CI 0.42 to 1.17; *p* < 0.001). The Heterogeneity among trials was substantial and significant (I^2^ = 71%, *p* = 0.032). One possible source of Heterogeneity was the Electrode positions. We reperformed meta-analysis considering only two studies with the same Electrode positions (Tecchio et al.^36^and Cancelli et al.^37^). The results indicated a significant large effect of Real (ES = 0.98, 95% CI 0.69 to 1.27; *p* < 0.001). The Heterogeneity was not significant (I^2^ = 0%, *p* = 0.503) (Table 8, Fig. 8).

**Table 8:** Fatigue. Real.

Study	PY	Study designed	Scale	n	Mean pre	SD pre	Mean post	SD post	Baseline-post mean change	SD Diff	Effect size (SMD)	SE of effect size
Tecchio et al	2015	Crossover	MFIS	13	42	8	31	12	11	7	0.9	0.19
Cancelli et al	2018	Crossover	MFIS	10	53	10	28	19	25	13	1.1	0.23
Ferrucci et al	2014	Crossover	FIS	23	118	41	100	34	19	22	0.5	0.12
**Pooled analysis**											**0.80**	**0.19**

PY = Publication Year. SMD = Standardized Mean Difference. SE = Standard Error.

##### Ms Fatigue–Real vs Sham effect

All the studies about fatigue in multiple sclerosis patients were crossover studies. We estimate the mean difference, SMD, between the mean value post Sham and post Real.

The results showed that the Real tDCS had not a significant effect compared to Sham, the pooled standardized mean difference of mFIS observed after tDCS was 0.34 standard deviation (SD) lower than the fatigue observed after Sham (95% CI = 0.24 to 0.92; *p* = 0.247) (Table 9). Heterogeneity was substantial and significant (I^2^ = 71.7%, *p* = 0.029). There were only three small studies and it is difficult to evaluate the funnel plot.

**Figure 9:**
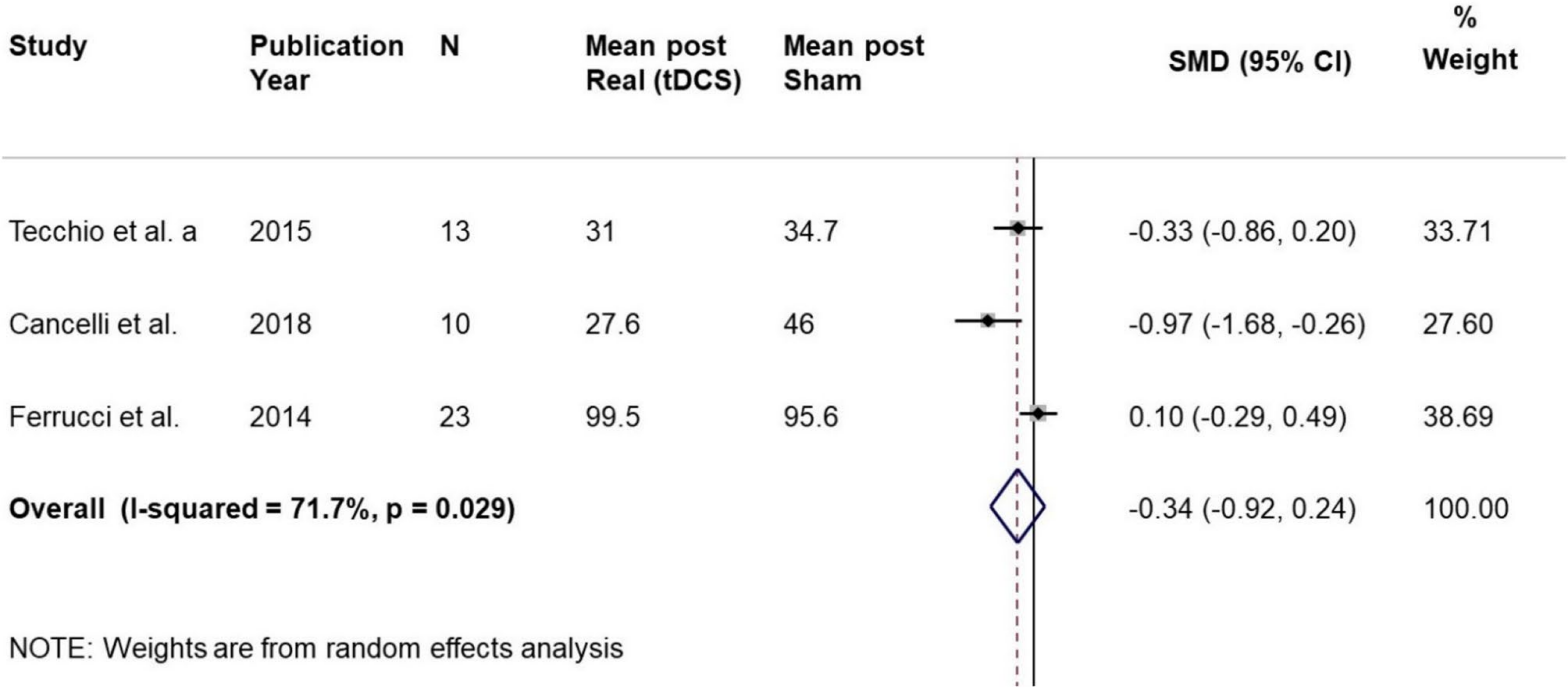
MS Fatigue. Real Vs Sham. Forest plot of meta-analysis results considering all studies. The diamond represents the pooled standardized mean difference (SMD, dashed red vertical line) and its 95% confidence interval; vertical solid dark line is the line of equivalence. The estimates for each study and their 95% confidence intervals are represented by a box with whiskers, the dimension of grey box is proportional to the precision of the study.

We reperformed meta-analysis considering only two studies with the same Electrode positions (Tecchio et al.^36^ and Cancelli et al.^37^). The results showed a marginally significant moderate effect of Real (tDCS) compared to that of Sham (ES = 0.61, 95% CI 0.02 to 1.23; *p* = 0.056). The Heterogeneity was not significant (I^2^ = 50.6%, *p* = 0.155).

**Table 9:** Fatigue. Real Vs Sham.

Study	PY	Study designed	n	Mean post Real	SD post Real	Mean post Sham	SD post Sham	Effect Size (SMD)	SE
Tecchio et al	2015	Crossover	13	31	12	35	10	0.33	0.27
Cancelli et al	2018	Crossover	10	28	19	46	19	0.97	0.36
Ferrucci et al	2014	Crossover	23	100	34	96	42	0.10	0.20
**Pooled analysis**								**0.34**	**0.30**

PY = Publication Year. SMD = Standardized Mean Difference. SE = Standard Error.

### PICO variables for tDCS against MS fatigue

Given that the few Class 1 RCTs involved small patients populations, we deduced the suggested parameters (Table 10) aware of the need of confirmation in larger groups.

**Table 10:** Parameters. Fatigue.

Study	Outcome	Population	Intervention						
			Electrode position		Stimulation intensity			Duration	
			Anode	Cathode	Electrode size	Ci	Csd	Daily	Days
Tecchio 2015	mFIS	EDSS ≤ 3 mFIS > 38 BDI < 19 No clinical relapse	S1	Oz	35/70	1.5	0.04	15	5
Cancelli 2018	mFIS	EDSS ≤ 2 mFIS > 35 BDI < 19 No clinical relapse	S1	Oz	35/70	1.5	0.04	15	5
Ferrucci 2014	FIS	EDSS 0–6.5 mFIS > 45	C3 + C4	Right deltoid	(35 + 35)/35	1.5	0.02	15	5

PICO variables for tDCS against MS fatigue, see the legend of Table 12 Depression.

The MS patients populations who benefitted by tDCS suffered by severe fatigue symptoms, as quantified by Modified Fatigue Impact Scale (mFIS) > 35, which appears to be a sufficient cut-off, since positive effects of the treatment were observed from this inclusion threshold. All cross-over studies involved small populations, and the results suggest that the inclusion of minimal to moderate clinical severity (Expanded Disability Status Scale, EDSS < 3.5) is opportune at the present stage instead of offering the tDCS treatment to patients in a wider range of disease-related impairment. While it will be relevant to assess the efficacy in presence of increasing disability in larger populations, it is of note that MS is typically accompanied by severe fatigue even in the absence of other disabling symptoms, thus a treatment efficacious against fatigue becomes decisive for the patient's quality of life.

Present data, corroborated by a recent wide-population multi-centre RCT^39^, suggest mFIS as proper outcome measure, sufficient with its 21-items to sense the induced variations, with no necessity to collect the longer 40-items Fatigue Impact Scale (FIS).

When defining the most efficacious tDCS intervention parameters, we observe a huge difference in efficacy when the anode targeted the bilateral whole-body primary somatosensory cortex, S1^36,37^ instead of the left and right hand sensorimotor regions^25^ where Real tDCS had not a significant effect compared to Sham. For the cathode, it appears appropriate to enlarge the surface area to minimize the effects in the reference area selected in occipital cortex to focus the induced current in post-central regions (Fig. 6). Another source of difference with Ferrucci et al. study^25^ could be the extra-cephalic reference, but this choice to minimize the undesired effects under the cathode is frequently used, with positive results in other cases.

In relation to the current intensity, the lower efficacy in Ferrucci et al. study^25^ could be due to the half current superficial density delivered across the two electrodes centred on C3 and C4, each 35 cm^2^ study. Thus the suggested value is 0.04 mA / cm^2^.

In relation to the duration, 15 min per day for 5 days of treatment seem sufficient.

The result of the present meta-analysis of the Class 1 tDCS RCTs further strengthens the findings of a recent systematic review and meta-analysis focused on non-invasive brain stimulations against fatigue^40^, which indicated that short-term and long-term treatment effects were significant for tDCS, whereas real TMS and transcranial random noise stimulation were not superior to sham stimulation. Among the 11 tDCS RCTs, the strongest efficacy emerged targeting the bilateral whole body S1^35–37^.

Positive indications of tDCS efficacy against fatigue when targeting primary somatosensory cortex suggest that positive effects can be induced also in dystonia. In fact, although dystonia is a heterogeneous neurological condition which manifests mainly as a movement disorder, from a physiological point of view the core generating mechanism is an abnormal sensorimotor integration^41^. Abnormalities can be found at multiple level such as a loss of inhibition, sensory disfunction and alterations in synaptic plasticity at different levels of sensorimotor circuit ^42,43^. Although there are no Class 1 RCTs available to support a consistent use of tDCS in dystonia, there are seminal results of efficacy coming from studies targeting specific dystonic conditions^24^. We can speculate about future perspectives in the treatment of dystonia by means of electroceutical interventions aiming at restoring the pathological functional unbalances, capitalizing on the results coming from available and sound RCTs that in counteracting efficaciously fatigue are able to rebalance altered mechanisms similar to those found in dystonia within the sensorimotor circuit.

### Pain

#### Quantitative analysis

A single study^44^ on 18 people suffering by back pain either bilaterally of with a monolateral prevalence satisfied the Class 1 criteria. Tables 11, 12, 13 reportsthe pre- and post- Sham and Real treatments values of the outcome measure and the differential effect of Real with respect to Sham.

**Table 11:** Pain. Sham.

Study	PY	Study designed	Scale	N	Mean pre	SD pre	Mean post	SD post	Mean Diff	SD Diff	SD within*	Effect Size (SMD)	SE
Straudi et al	2018	Double-blinded randomized controlled	VAS	17	50.3	24.4	41.5	24.2	- 8.8	29.2	24.30	0.36	0.30

*the correlation, r = 0.28, between pre-post data were extracted from the variance of the difference. PY = Publication Year SD = Standard Deviation. SMD = Standardized Mean Difference. SE = Standard Error.

**Table 12:** Pain. Real.

Study	PY	Study designed	Scale	N	Mean pre	SD pre	Mean post	SD post	Mean Diff	SD Diff	SD within*	Effect Size (SMD)	SE
Straudi et al	2018	Double-blinded randomized controlled	VAS	18	55.7	18.3	38.8	23.4	- 16.90	20.4	21.37	0.79	0.26

*the correlation, r = 0.547, between pre-post data were extracted from the variance of the difference. PY = Publication Year SD = Standard Deviation. SMD = Standardized Mean Difference. SE = Standard Error.

**Table 13:** Pain. Real Vs Sham.

Study	PY	Study designed	Scale	Diff between Baseline-post mean change	SD within	Effect Size (SMD)	SE
Straudi et al	2018	Double-blinded randomized controlled	VAS	- 8.1	25.06	0.32	0.34

PY = Publication Year. SMD = Standardized Mean Difference. SE = Standard Error.

#### PICO variables for tDCS against pain

Straudi et al.^44^, the only study against pain matching the Class 1 requirements, involved a group of people suffering by back pain, without comorbidities, at a mean level of 5.6 ± 1.8 cm of the Visual Analogue Scale (VAS) for pain intensity with maximum 10 cm. The VAS is proposed as a proper outcome measure. In dependence on the prevalence of symptoms, the anode was placed on the primary motor cortex (M1) of the dominant hemisphere if pain was central in the back or bilateral, or on contralateral M1 if pain irradiated to one side. The cathode was positioned on the supraorbital area contralateral to the anode. We formulate a doubt about the choice of centering the electrode on C3/C4, corresponding to the hand representation, instead of a more central position corresponding to lower body representation. For cathodal position, electrode size, current intensity and stimulation duration refer to the Table 14.

**Table 14:** Parameters. Pain.

Study	Outcome	Population	Intervention						
			Electrode position		Stimulation intensity			Duration	
			Anode	Cathode	Electrode size	Ci	Csd	Daily	Days
Straudi 2018	VAS for pain intensity	18–75 years old non-specific chronic low back pain VAS > 20 mm (over 100 mm)	C3/C4	Contra-lateral Supraobital area	35	2	0.057	20	5

PICO variables for tDCS against pain, see the legend of Table 12 Depression.

#### Fibromyalgia

In our query, four studies reported positive results of tDCS intervention against fibromyalgia symptoms. Since applying the PICO model no clear diagnostic criteria exist for fibromyalgia, we excluded this condition from the quantitative analysis and we report the studies in a descriptive manner.

Two studies focused on the primary symptom of this pathology, pain. Riberto et al.^45^ applied tDCS over left M1 (2 mA, 20 min, 1 session per week, 10 weeks) combined with a rehabilitation program. They observed a reduced impact of pain on the quality of life after Real vs Sham tDCS but not differential effect on pain intensity, depression and anxiety.

To et al.^46^ applied tDCS (1.5 mA, 20 min, 2 sessions per week, 4 weeks) comparing the efficacy of two bilateral montages, occipital (C2 dermatomes) and dlPFC (F3 and F4), in reducing fibromyalgia-related pain and fatigue. They observed a significant reduction of pain but not fatigue when targeting the C2 dermatomes region and a significant reduction of both pain and fatigue when targeting bilateral DLPFC.

Other two studies focused on secondary cognitive aspects related to fibromyalgia, as attention and memory performance.

Santos et al.^47^ combined tDCS on left DLPFC (1 mA, 20 min, 8 session for 8 consecutive days) with working memory training, obtaining an improvement of immediate memory capacity and verbal fluency. Moreover, Silva et al.^48^ combining tDCS with Go/No-Go task, reported an improvement of attention performance as primary outcome together with an improvement of pain threshold considered as secondary outcome.

Overall, these results from Class 1 RCTs integrate with promising evidence of tDCS efficacy in the treatment of pain and cognitive dysfunctions.

#### Addiction

Five Class1 RCTs used tDCS against addiction behaviors. We review the results without executing the meta-analysis since it was not possible to pool them in a common statistic, either for absence of indication of a primary outcome among multiple variables to quantify the effect, or for inhomogeneity of the outcome (e.g., relapse rate vs. number of smoked cigarettes).

Against alcohol addiction, Klauss et al.^49^ targeted right dlPFC by anode (2 mA, 13 min, twice per day, 5 days) against left dlPFC and reported a significant reduction in relapse rate up to six months. Den Uyl et al.^50^ tested the efficacy of tDCS, anode over left dlPFC and cathode over right dlPFC, in enhancing the effect of a Cognitive Bias Modification (CBM) in patients with alcohol addiction. Despite the results could not isolate the effect of tDCS intervention, tDCS combined with CBM showed no effect on alcohol approach bias, craving or time to relapse and only a trend of significance on reduction in probability of relapse.

Two other studies targeted smoking behavior with similar montage over right or left DLPFC.

Mondino et al.^51^ delivered tDCS (2 mA, 20 min, twice per day, 5 days, anode F4) focusing on self-reported smoking intake and brain activity via fMRI. Despite no effect of tDCS on smoking intake, they found a significant reduction in smoking craving and increased brain reactivity of the right posterior cingulate area to smoking cues. Conversely, Falcone et al.^52^ (anode F3, 1–2 mA, 20 min, 3 times per week, 1 week) observed no effect of tDCS on abstinence or number of days of abstinence or change in daily smoking rates nor on latency to smoke or number of cigarettes smoked.

There are indications of efficacy as demonstrated by Batista et al.^53^ about cocaine addiction. By targeting right DLPFC (2 mA, 20 min, 5 days, 3 weeks) they found an improvement in craving scores, anxiety and overall perception of quality of life with significant effects even after weeks.

We note that looking for tDCS effects against addition, the complication emerges about the interaction with a wide range of systemic and multi-organ consequences as the effect of the abused substance.

#### Estimation of Sham effect

A further contribute of this quantitative review is the estimation of Sham effect in trials planned to assess the efficacy of tDCS.

**Figure 10:**
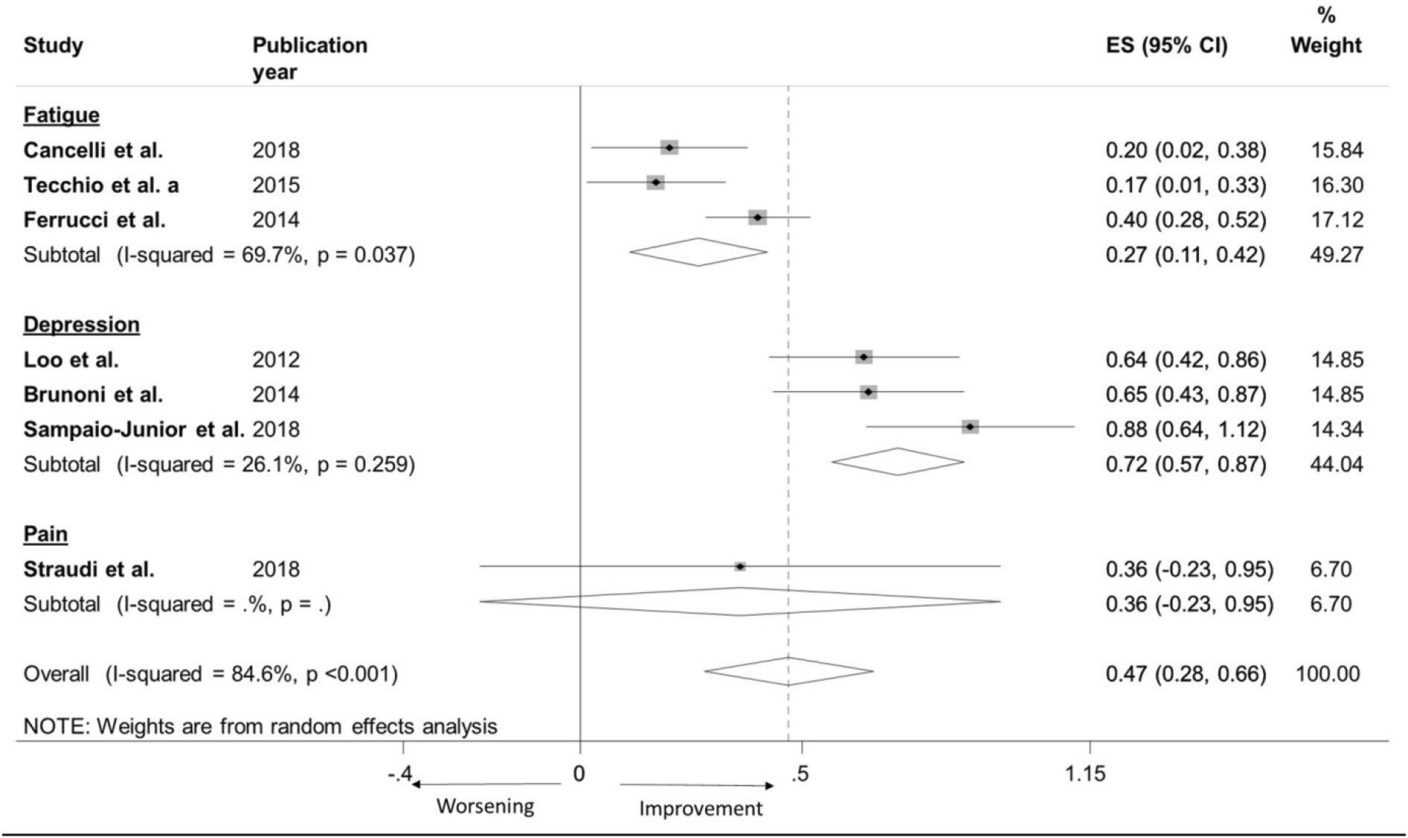
Estimation of Sham effect. (**a**) Forest plot of meta-analysis results considering all studies by pathology (MS fatigue, Depression, Pain). The diamond represents the pooled standardized mean difference (SMD, dashed red vertical line) and its 95% confidence interval; vertical solid dark line is the line of equivalence. The estimates for each study and their 95% confidence intervals are represented by a box with whiskers, the dimension of grey box is proportional to the precision of the study. Note: ES = Effect Size. CI = Confidence Interval.

According to Fig. 10, Sham effect resulted clearly dependent on pathology/symptom. After excluding pain (Sham effect was estimable only in one paper and with high level of imprecision), it is evident (test for subgroup differences: I2 = 94%, Chi^2^(1) = 16.75, *p* < 0.001) that Sham effect was markedly different in fatigue and depression. We estimated that Sham effect size in fatigue is 0.27 (a small effect) and 0.72 in depression (a quite large effect).

In future RCTs aiming at demonstrating an effect of a Real stimulation, researchers could consider these as “first guess” of the control Sham condition. Table 15 presents different scenarios. On the basis of our estimation of Sham effect on fatigue (standardized Effect Size, sES = 0.27), in order to reach a 90% probability (power) of recognizing as statistically significant (at two-sided alpha level set at 0.05) an increase of Real stimulation up to a medium effect size (sES = 0.50), a sample size of almost 800 patients will need for a two-arm parallel design. Such number decreases to about 200 patients in case of a one-sample design, thus without the recruitment of patients to be assigned to control Sham treatment. A further decrease of sample size could be obtained with a cross-over design (n = 122), for which the correlation between pre-post Sham and pre-post Real changes was assumed equal to r = 0.7. Such correlation would imply that about 50% of changes’ variance observed after Real stimulation could be accounted for by changes observed after Sham stimulation. We also indicated the sample size estimate in the case of the large Real stimulation effect resulting from the present meta-analysis (sES = 0.98), which corresponds to much smaller population dimensions.

**Table 15:** Sham Power Analysis.

Symptom/pathology	Two-sided alpha	Power (1-beta)	Expected sham-effect (from this meta-analysis)	Effect size of Real stimulation (minimal clinically relevant difference)	Target effect size of Real stimulation	Design	Sample size
Fatigue	0.05	0.9	0.27	0.5	Medium	one-sample	201
						parallel two-samples	399 + 399
						cross-over*	122
				0.8	Large	one-sample	40
						parallel two-samples	76 + 76
						cross-over*	25
				0.98	Based on this meta-analysis	one-sample	23
						parallel two-samples	43 + 43
						cross-over*	15
Depression	0.05	0.9	0.72	0.8	Large	one-sample	1644
						parallel two-samples	3285 + 3285
						cross-over*	987
				1	Very large	one-sample	136
						parallel two-samples	270 + 270
						cross-over*	83
				1.79	Based on this meta-analysis	one-sample	12
						parallel two-samples	20 + 20
						cross-over*	8

Sample size estimation for different effect size of Real stimulation vs. estimated effect size of Sham stimulation, according to the present meta-analysis. Sham effect size in fatigue resulted = 0.27, thus effect size of Real stimulation was set at 0.5 (medium) and 0.8 (large). Sham effect size in depression resulted = 0.72, thus effect size of Real stimulation was set at 0.8 (large) and 1.0 (very large). In addition, the effect size of Real stimulation (estimated with this meta-analysis) was considered in order to indicate that effects of this magnitude (and thus also smaller) could be considered realistic. * a correlation equal to 0.7 is assumed.

Following this logic, Table 15 reports the appropriate sample size for different effect size of Real stimulation for depression. To be noted that, if it is considered clinically relevant an effect size of 0.8 for Real stimulation on depression (that corresponds to a large effect size according to Cohen’s convention), a huge sample size should be recruited for a parallel design (about 7500 patients). This was due to the high estimated sham-effect for this condition. In other terms, a future trial should face the probable large effect of sham stimulation and, thus, Real stimulation should be able to induce an even higher benefit on depression scales. Nevertheless, the observed effects in depression were huge after Real stimulation, with the meta-analysis estimation of ES = 1.79. In addition, more efficient design, such cross-over, are recommended, provided that a good correlation between changes (within-subjects dependency) could be assumed.

Aware of the small populations involved in this sample sizes’ estimate, we pave the way for assessing the Sham effect for future studies, where the assessment of Real efficacy will get rid of the need of employing wide efforts and long times for patients and experimenters in the Sham assessment. We note that in depression the Sham effect was much higher than in fatigue. A strong placebo effect in depression was also found in pharmacological treatment RCTs, where a high efficacy of the antidepressant medication was almost as strong as placebo's^54^. The Authors of that wide review pose the provocative statement suggesting as preferable the placebo to the pharmacological treatments to avoid the documented severe side effects. In multiple sclerosis, a condition where therapeutic drugs contribute to fatigue generation^55,56^, the pharmacological treatments of the fatigue symptom are poorly effective^57^, thus stimulating the use of alternative substances, which also reach poor efficacy (ES = 0.04,^58^), and similarly to depression, the ameliorations to placebo can be similar to that to effective therapy^39,59^. These findings support the development of electroceutical solutions, almost free of side effects, which produce as presently reviewed relevant efficacies, open to personalization to further enhance the amelioration for individual patients^60^.

### rTMS as opposed to tDCS

While some scientists^61,62^ raised skepticism on the effectiveness of tDCS ((as opposed to the validity of rTMS)) we believe that not only methodological and conceptual errors invalidate these general conclusions (e.g.,^63,64^; See also^65^) but the core technological and physical differences in the generated stimulation make rTMS preferable when focused and high intensity stimulation are required while tDCS can be used when targets are wider cortical areas and even small currents are effective.

### Study limits

We executed a quantitative review, aware that for a systematic review the inclusion of all search engines would be required. However, we decided to query only the PubMed repository considering that it represents the most comprehensive archive of biomedical and life sciences journal literature, thus expecting that it fully reports papers on RCTs. Notably, other sources like WebOfScience and Scopus typically include abstracts and conference communications in addition to the fields represented in PubMed of the covered scientific themes.

Quality assessment of all RCTs found by the query was carefully executed verifying each GRADE criteria that define Class 1 studies (listed in Table 2), by three experts providing multidisciplinary expertise (Biostatistician, Psychologist, Philosopher with a PhD in Neuroscience). Since the final included studies were in a small number for each pathology, a Funnel plot was reported when possible while no statistical test to evaluate publication bias was applicable.

## Conclusions

Our study shows that RCTs employing tDCS are growing in number and that at least some of them can be classified in Class 1, supporting good reliability of the observed clinical efficacy in pathologies related to neuronal activity imbalances. Our quantitative review points towards a treatment recommendation according to the GRADE classification criteria between moderate and high for depression, fatigue in MS and pain (Fig. 1), thus indicating tDCS treatment to be a promising tool in these cases for which we also provided the treatment parameters within the PICO model. Furthermore, we meta-analysed the Sham effect size and provided the consequent sample size quantifications for future study designs where the Sham effect is known a-priori without experimental execution. Forthcoming research is necessary to strengthen the tDCS intervention GRADE recommendation by enlarging the samples and paving the way to the intervention personalization.

## Data Availability

The data that support the findings of this study are available from the corresponding author, FT, upon reasonable request.
